# An efficient and successful outcome after haematopoietic stem cell transplantation in a patient with an LPS-responsive beige-like anchor gene mutation

**DOI:** 10.3389/fped.2024.1420118

**Published:** 2024-07-23

**Authors:** Cen Shen, Luying Zhang, Yan Meng, Lu Yang, Wenli He, Xiaoying Lei, Lina Zhou, Yunfei An, Ying Dou

**Affiliations:** ^1^ Department of Hematological Oncology, Children’s Hospital of Chongqing Medical University, National Clinical Research Center for Child Health and Disorders, Ministry of Education Key Laboratory of Child Development and Disorders, Chongqing, China; ^2^ Chongqing Key Laboratory of Child Rare Diseases in Infection and Immunity, Chongqing, China; ^3^ Department of Immunology, Children’s Hospital of Chongqing Medical University, National Clinical Research Center for Child Health and Disorders, Ministry of Education Key Laboratory of Child Development and Disorders, Chongqing, China

**Keywords:** LRBA mutation, haematopoietic stem cell transplantation, neurological change, infection, graft-versus-host disease

## Abstract

Lipopolysaccharide (LPS)-responsive beige ankyrin (LRBA) gene mutations were first reported as the cause of immunodeficiency syndromes and autoimmunity in 2012. The majority of LRBA patients have multiple organ system involvement and a complex clinical phenotype. Herein we present a comprehensive account on the disease progression and transplantation procedure in a patient with LRBA deficiency who exhibited progressive autoimmune disease symptoms along with recurrent pulmonary infections since the age of 6 years old. Despite receiving abatacept therapy and immunoglobulin replacement treatments to manage the symptoms, but the symptoms still progressed. Therefore, nine years after disease onset, patients were treated with allogeneic haematopoietic stem cell transplantation (allo-HSCT). The patient experienced acute and chronic graft-versus-host disease (GVHD) and recurrent infections after transplantation. During one and a half years of follow-up, we found that allogeneic haematopoietic stem cell transplantation can relieve the symptoms of autoimmune disease in patients with LRBA deficiency, and marked clinical improvement and recovery of immune function were observed following stem cell transplantation.

## 1 Introduction

CTLA-4 is an immune checkpoint molecule predominantly expressed on the surface of *T* cells (1). LRBA proteins regulate the intracellular trafficking and storage of CTLA4, facilitating its rapid mobilization to the cell surface for effective suppression of hyperactivation (2). The main clinical manifestations of LRBA deficiency include recurrent respiratory infections, lymphoproliferation, and immune dysregulation characterized by inflammatory bowel disease, autoimmune cytopenia, and autoimmune hemolytic anemia (3). Additionally, patients with LRBA deficiency often develop bronchiectasis and interstitial lung diseases following recurrent infections (4, 5). Currently, abatacept (a CTLA4-immunoglobulin fusion drug) has shown significant and sustained improvement in patients with LRBA deficiency (2, 6). While allogeneic hematopoietic stem cell transplantation (HSCT) is a standard treatment for various primary immunodeficiencies (PIDs), there are no established guidelines regarding HSCT in patients with autoimmune diseases. In this study, we present the clinical features and treatments of a patient diagnosed with LRBA deficiency. Hematopoietic stem cell transplantation has long-term efficacy in the treatment of patients with LRBA deficiency.

## 2 Method

### 2.1 Western blot

Isolated peripheral blood mononuclear cells (PBMCs) were stimulated with 10 ng/µl PHA for 48 h. Protein was extracted in RIPA and PMSF buffer. Protein extracts underwent electrophoresis on a 6% concentration polyacrylamide gel and were subsequently transferred to nitrocellulose membranes. The membranes were initially incubated in a blocking buffer followed by incubation with specific antibodies. For this study, LRBA antibody (1:1,000, Abcam, ab191174), DOCK8 antibody (1:5,000, Abcam, ab175208), and HRP-conjugated goat anti-mouse secondary antibody (1:1,000, Beyotime, A0216) were employed.”

### 2.2 Flow cytometry

PBMCs were directly stained with the surface markers CD4-PE-Cy7, CD25-BV421, and CD45RA-FITC, followed by fixation/permeabilization (BD Biosciences) according to the manufacturer's instructions. Then, the cells were stained with fluorochrome-conjugated antibodies FOXP3-PE and CTIA4-APC. Analyses were performed using BD Celesta.

## 3 Results

### 3.1 Before transplantation

#### 3.1.1 Clinical manifestations

**Figure 1:**
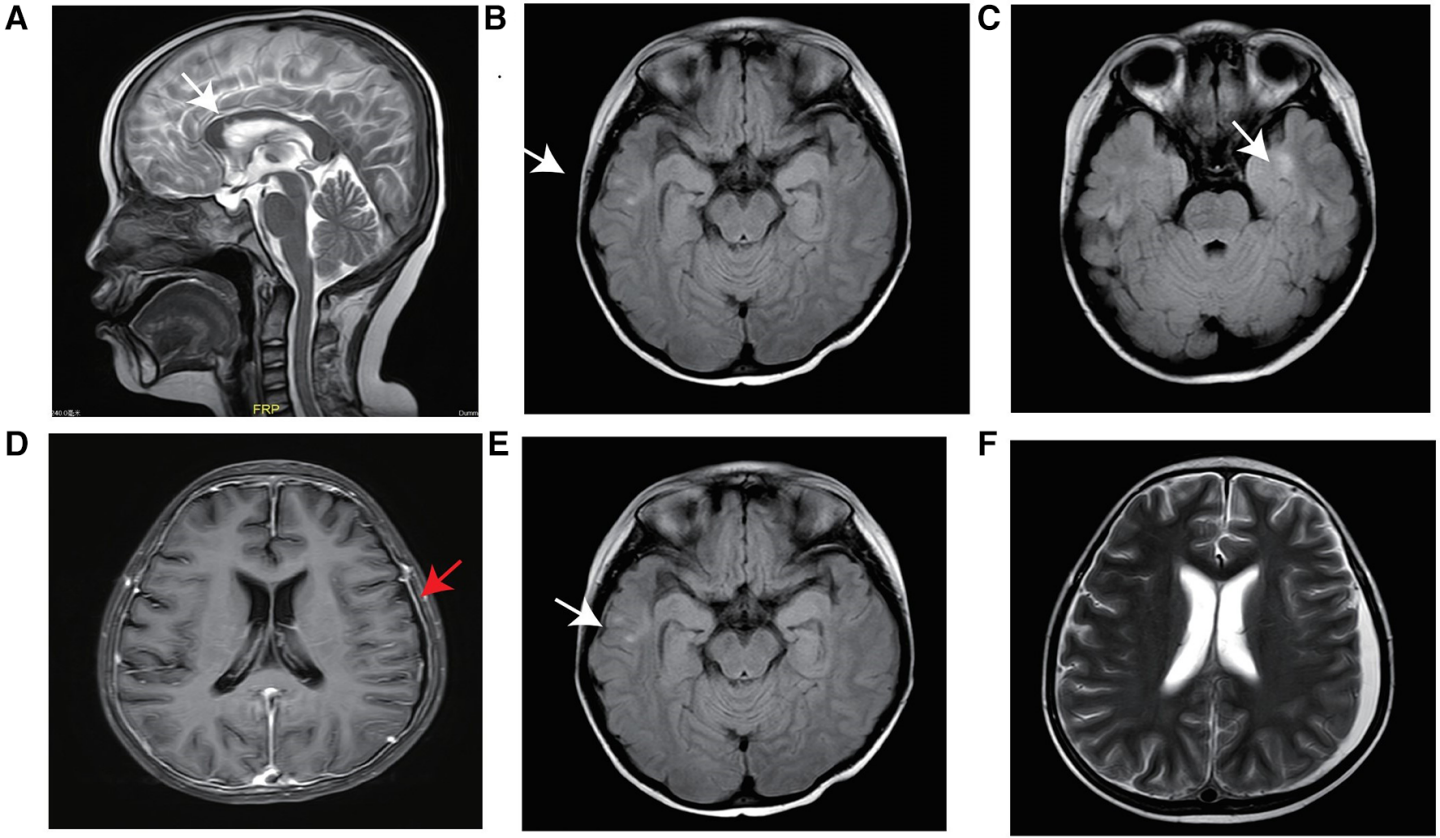
MRI of the brain. Abnormal signals from the corpus callosum body to the bilateral frontal ventricle and bilateral temporal lobe before transplantation (white arrow) (**A**–**C**), linear enhancement of the bilateral frontoparietal and occipital meninges after HSCT (red arrow) (**D**), abnormal signals (**E**), and a small amount of new subdural effusion in the left frontotemporal parietal region (**F**).

A 6-year-old boy presented with autoimmune haemolytic anaemia (AIHA) and thrombocytopenia. Following treatment with immunosuppressive agents, no further episodes of AIHA have been observed. In addition to the manifestation of Evans syndrome, the patient also exhibited recurrent infection and hypogammaglobulinemia after reaching the age of 10 years, especially Invasive pulmonary fungal infection. The emergence of recurrent cough and expectoration suggested pulmonary infection. Despite receiving multiple courses of antibiotics without sustained improvement, sputum smear examination revealed the presence of a small number of spores and the (1,3)-β-D glucan test yielded positive results, further indicating potential fungal infection. The patient was initially treated with oral prednisolone and received regular monthly immunoglobulin replacement. The growth and development in patients suffering from severe malnutrition were found to be below 3 SDs for children of similar age and sex after reaching 10 years old. At 13 years of age, lung CT revealed fibrotic lesions in the lower lobes of both lungs. At the same stage, abdominal ultrasonography revealed hepatosplenomegaly suggesting severe liver fibrosis possibly due to autoimmune hepatitis. In addition, brain MRI showed abnormal signals in the bilateral temporal lobe, right parietal lobe and corpus callosum without any evidence of infection (Figures 1A–C). Other clinical manifestations included interstitial kidney injury and hypokalaemia.

**Figure 2:**
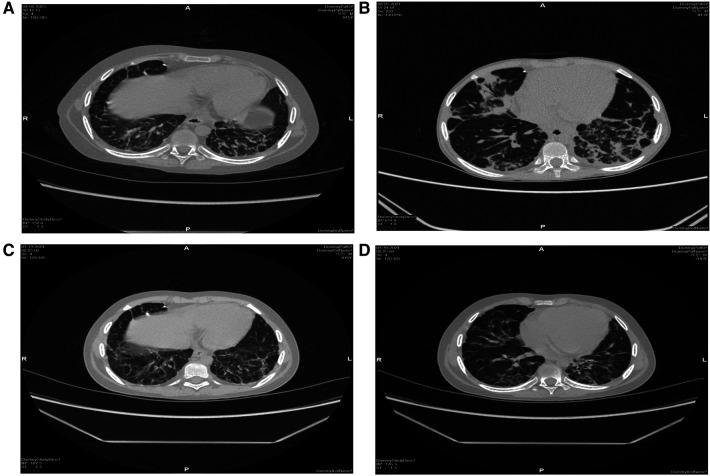
High-resolution CT scan of the lungs. Before transplantation, bilateral pulmonary fibrosis with segmental consolidation in the left lower lobe (**A**,**B**), consolidation was significantly absorbed, and fibrosis improved after transplantation (**C**,**D**).

Considering that LRBA deficiency may contribute to autoimmune disorders in patients, targeted therapy with subcutaneous injection of abatacept at a dosage of 125 mg/w was administered for 5 months prior to transplantation. However, despite the combination of abatacept and immunosuppressive therapy, complete prevention of disease progression was not achieved. Although examination results indicated reduced liver and spleen sizes in the patient, recurrent pulmonary infections persisted along with persistently low levels of immunoglobulins and B-cells (Figures 2A,B).

On the eve of transplantation, the child developed symptoms of septic shock due to sepsis. Following anti-shock treatment, improvements were observed in blood pressure and mental state before proceeding with immediate hematopoietic stem cell transplantation. Pre-transplantation laboratory findings are presented in Table 1.

**Table 1:** Laboratory workup of the patient with lipopolysaccharide-responsive beige-like anchor deficiency.

Lab results	Pre-transplantation	Post-Transplantation(1Y6M)	Normal range
WBC	4.98	4.4	4.1–11.0 (10^9^ /L)
RBC	5.21	4.19	4.6–6.0 (10^12^ cells/L)
PLT	189	178	100–407 (10^9^ cells/L)
NE	57.7	56.9	37–77 (%)
LYM	33.5	38.2	17–54 (%)
Total bilirubin	6.8	8.8	2.68–35.26 (umol/L)
Direct bilirubin	0	0	0–10 (umol/L)
Indirect bilirubin	4	6.4	0–18.67 (umol/L)
Total protein	43⬇	71.6	64.6–84.7 (g/L)
Albumin	22.6⬇	41.4⬇	42.5–54.8 (g/L)
Globulin	20.4	30.2	15.3–35.0 (g/L)
ALT	55⬆	22	11.4–45.4 (U/L)
AST	96⬆	30	13.9–35.3 (U/L)
ALP	175	198	62.0–334.5 (U/L)
Glutamyl transpeptidase	289⬆	15	10.0–27.9 (U/L)
LDH	290⬆	255	105–257 (U/L)
Cholinesterase	6,328	7,948	5,900–12,220 (U/L)
Urea	3.34	2.69	2.1–7.1 (mmol/L)
Creatinine	42	21⬇	30.5–103.5 (umol/L)
Uric acid	312	245	185.9–465.7 (umol/L)

#### 3.1.2 Immunological manifestations

LRBA gene mutations were confirmed through whole exome sequencing. The patient harbors a compound heterozygous mutation, c.4801C>T (p.Arg1601*), located in exon 30, which constitutes a nonsense mutation inherited from the father. The other mutation, c.4239delT, represents a deletion variant originating from the mother. Functional validation was performed by western blotting, revealing an absence of LRBA expression and reduced CTLA4 expression in the patient (Figures 4A,C). Notably, there was a significant decrease in the proportion of naive CD4+ *T* cells, particularly CD4 CM cells; meanwhile, the proportion of CD8 CM cells among total CD8+ *T* cells increased. Due to their low abundance for testing purposes, B cell proportions could not be determined (Table 2). It is worth mentioning that the patient's immunoglobulin level remained consistently low throughout this study period (Figure 6E), necessitating regular administration of immunoglobulin.

**Table 2:** Fine classification of immune cells.

Lab results	Relative number (%)			Absolute number (cells/μl)		
	Pre-transplantation	Post-transplantation(1Y6M)	Normal range	Pre-transplantation	Post-transplantation(1Y6M)	Normal range
*T* cell (CD3+)	91.2⬆	80.7⬆	56.84–75.02	11,541.6⬆	1,354.9	1,184–2,144
CD8+ *T* cell (CD3 + CD8+)	30.06	53.8⬆	21.91–36.80	508	904.0	489–1,009
CD8 Naïve (CD8 + CD45RA + CD27+）	53.1	18.5⬇	35.34–72.32	269.8	167.2⬇	231–568
CD8 TEMRA (CD8 + CD45RA + CD27−）	9.5	24.6	5.08–31.24	48.2	222.4	29–269
CD8 CM (CD8 + CD45RA−CD27+)	37.1⬆	32.9⬆	10.96–31.00	188.5	297.4⬆	74–228
CD8 EM (CD8 + CD45RA−CD27−）	0.3⬇	23.9⬆	2.38–15.84	1.3⬇	216.1⬆	16–109
CD4+ *T* cell (CD3 + CD4+)	49.5⬆	23.9	22.25–39.00	835.7	401.9	522–1,084
CD4 Naïve (CD4 + CD45RA + CD27+）	9.7⬇	27.1	39.50–66.26	81.3⬇	108.9⬇	230–627
CD4 TEMRA (CD4 + CD45RA + CD27−）	5.2⬆	5.3⬆	0.00–1.54	43.8⬆	21.3⬆	0–12
CD4 CM (CD8 + CD45RA−CD27+)	77.2⬆	34.9	25.34–49.90	645.2⬆	140.2⬇	182–403
CD4 EM (CD8 + CD45RA−CD27−）	7.9	32.7⬆	4.68–15.70	65.9	131.4	29–117
TCRαβ + DNT/T	1.4	0.3⬇	0.61–2.31	21.5	4.7⬇	12–37
γδ *T* cell	15.3	4.7⬇	6.55–20.28	235.9	63.7⬇	81–343
B cell (CD19+)	−	13.2	8.84–17.76	−	222.1	203–476
Memory B (CD19 + CD27 + IgD−)		3.1⬇	7.15–23.10		6.9⬇	20–86
Naïve B (CD19 + CD27−IgD+)		87.6⬆	53.78–78.64		194.6	116–347
Transitional B (CD19 + CD24 + CD38+)		34.9⬆	1.38–9.42		77.5⬆	4–37
Plasmablasts B (CD19 + CD24–CD38+)		0.9	0.49–7.06		2.0	1–23.0

CM, central memory; EM, effector memory.

### 3.2 HSCT

#### 3.2.1 HSCT process

Due to severe clinical complications and the inefficacy of drugs, haematopoietic stem cell transplantation has become a curative option. Fortunately, HLA (10/10)-matched unrelated donors were procured from the China Marrow Donor Program. The patient received mobilized peripheral blood cells containing 12.94 × 10^8 ^/kg MNC cells/kg and 7.47 × 10^6^ CD34+ cells/kg following reduced-intensity conditioning regimens, including fludarabine 45 mg/m^2^/d (D-7∼-4), BU 3 mg/kg/d (D-5∼-2), and ATG5 mg/kg/d (D-5∼-3) (Figure 5). The conditioning regimen was well tolerated without any signs of organ toxicity. Voriconazole was administered for fungal infection prophylaxis, Imipenem-Cilastatin Sodium combination drug for bacterial infection prevention, acyclovir for viral infection prevention, weekly gamma globulin infusion along with weekly monitoring of EBV and CMV-DNA quantification.

#### 3.2.2 Engraftment

Granulocyte recovery for haematopoietic reconstitution was continuously observed on Day 13 post-HSCT. Megakaryocyte recovery occurred on Day 15 post-HSCT. Methylprednisolone was administered on day 15 after transplantation to treat implantation syndrome (Figure 5). Stable chimerism, with T, B cells and whole blood showing complete donor engraftment (100% of donor), was observed starting from day +28.

### 3.3 After transplantation

#### 3.3.1 GVHD

The patient developed grade II acute gastrointestinal tract graft-versus-host disease (GVHD) on 18D, presenting as watery diarrhoea. Stool culture results were negative, and the patient had a daily stool volume exceeding 500 ml during the day. By increasing methylprednisolone dosage to 2 mg/kg/day and enhancing intravenous nutrition, significant improvement in GVHD was observed. The patient was discharged from the laminar flow ward after 35 days. However, six months post-transplantation, the patient experienced loose paste-like stools with occasional blood-streaked mucus. Stool cultures remained negative throughout this period and liver function examination revealed alanine transaminase (ALT) levels of 224 U/L and aspartate transaminase (AST) levels of 134 U/L. Thus leading to a diagnosis of chronic gastrointestinal tract and liver GVHD. Oral corticosteroid and mycophenolate mofetil doses were increased along with glutathione supplementation for liver protection alongside other symptomatic and supportive treatments. The patient improved and was discharged (Table 3).

**Table 3:** GVHD experienced by the patient after hematopoietic stem cell transplantation.

GVHD	Targeted organ	Score	Symptoms and clinical manifestations	Occurrence time	Therapy
aGVHD	Gastrointestinal tract	2	The patient presents with prominent diarrhea, characterized by profuse watery stools accompanied by scant fecal debris. Stool culture results are negative, and the daily stool volume exceeds 500 ml during the day.	18D	Refrain from administering cyclosporine and commence tacrolimus treatment, escalate the dosage of methylprednisolone to 2 mg/kg/day, incorporate omeprazole for acid suppression and gastric protection, administer parenteral nutrition support, and decrease oral food intake.
cGVHD	Gastrointestinal tract	1	The patient presents with 5–7 daily episodes of diarrhea, characterized by loose paste-like stools occasionally accompanied by blood-streaked mucus. Stool cultures yield negative results during this period, a noticeable improvement is observed after 12 days, with a reduction of bowel movements to 1–2 times per day.	6M	Intravenous administration of methylprednisolone at a dosage of 2 mg/kg/day, in combination with mycophenolate mofetil at a dosage of 45 mg/kg/day.
	Liver	2	ALT: 224 (U/L) AST: 134 (U/L)		

#### 3.3.2 Infection

**Figure 3:**
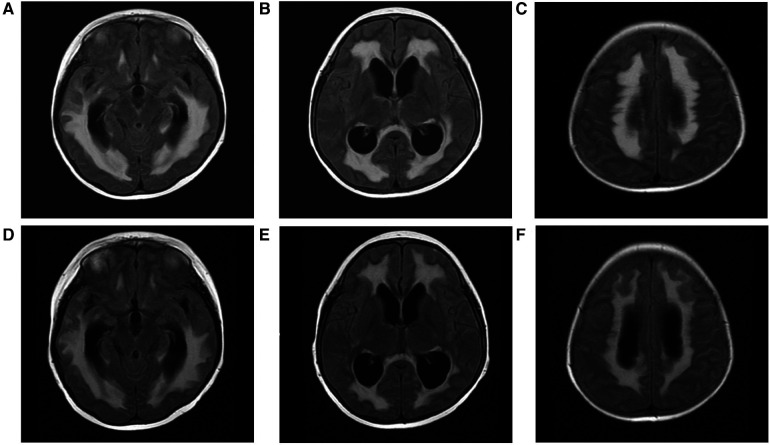
T2-FLAIR hyperintensity contrast-enhanced lesions, multiple abnormal signals in bilateral cerebral white matter after haematopoietic stem cell transplantation (**A**–**C**). Decreased area of white matter lesions after treatment (**D**–**F**).

The patient experienced recurrent nervous system, gastrointestinal tract and respiratory tract infections after transplantation. Two months after transplantation, the patient developed persistent consciousness disturbance and fell into a mild coma. Brain MRI revealed a slight increase in subdural effusion in the left frontotemporal region compared to pre-transplantation levels, along with linear enhancement observed bilaterally in the frontoparietal and occipital meninges (Figures 1D–F). Adenovirus PCR testing on cerebrospinal fluid yielded positive results. Four months later, the patient experienced recurring symptoms including headache, vomiting, and fatigue. Brain MRI showed multiple abnormal signals in bilateral white matter as well as linear enhancement of bilateral frontotemporal meninges (Figures 3A–C). Posttransplant leukoencephalopathy was diagnosed. Despite a negative cerebrospinal fluid (CSF) culture, the patient improved after treatment with antibiotics. During the next month, abdominal pain recurred accompanied by mucus and bloody stools. Abdominal CTA revealed bowel wall thickening and stool culture was positive for *Clostridium difficile* toxin B. Remission was achieved through treatment with metronidazole and vancomycin. Prior to transplantation, pulmonary fibrosis was predominant with no abnormalities detected during pulmonary function tests. Following transplantation though early stages witnessed recurrent pulmonary infections; however lung CT scans indicated significant improvement compared to pre-transplantation conditions (Figures 1C,D). One year post-transplantation there were no apparent signs of infection observed in the child while neurological examination also displayed no abnormalities (Figures 3D–F).

#### 3.3.3 Immunological recovery

**Figure 4:**
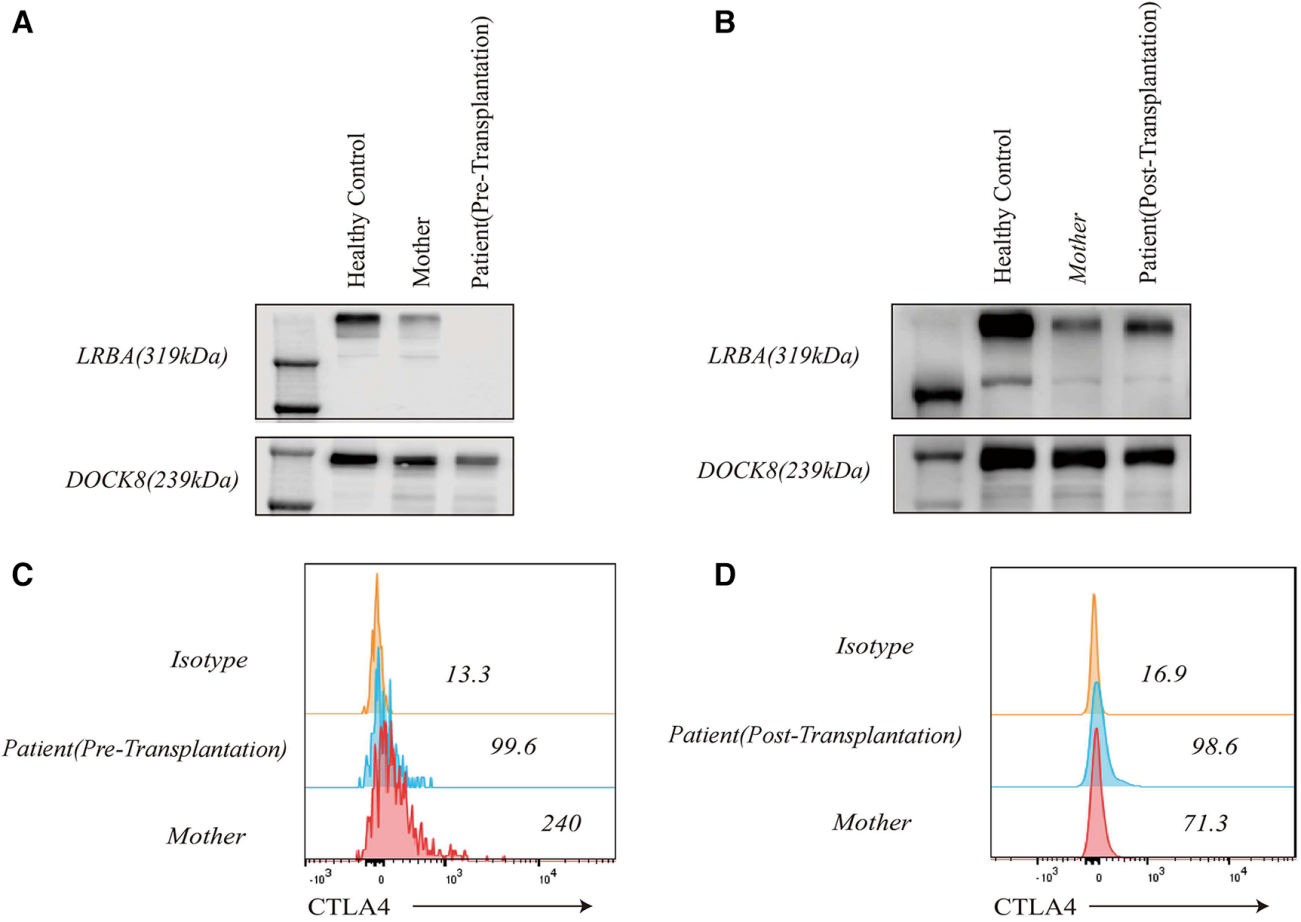
LRBA and CTLA4 expression levels in the patient, his clinically healthy mother and healthy control. LRBA expression was absent in the patient before transplantation (**A**), and CTLA4 expression was reduced (**C**) LRBA expression (**B**) and CTLA4 levels (**D**) normalized after haematopoietic stem cell transplantation.

LRBA protein and CTLA4 expression were detected at the ninth month after HSCT showing similarity to that in the patient's parents and healthy controls (Figures 4B,D). The patient's immune status was continuously monitored after transplantation. Although CD4+ *T*-cell counts were slightly low early after transplantation, they returned to normal levels. Reconstitution of CD8+ *T* cells began early after transplantation (Figures 6A,C) (Table 2). The patient's B cell counts remained low after transplantation, and gamma globulin was administered regularly. Fortunately, one and a half years after transplantation, the patient's B cells returned to normal levels, along with normalization of immunoglobulin levels. (Figures 6B,D) (Table 2). IVIG replacement therapy was discontinued at 1 year and 8 months after transplantation (Figure 6E).

**Figure 5:**
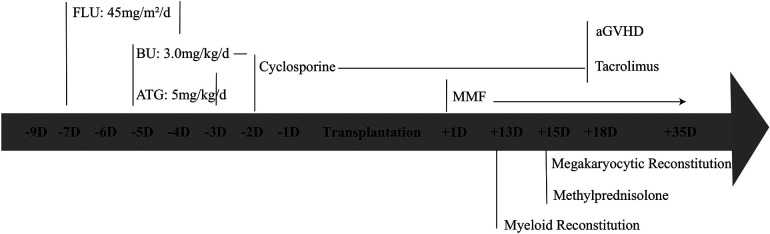
Elaboration on the transplantation procedure.

**Figure 6:**
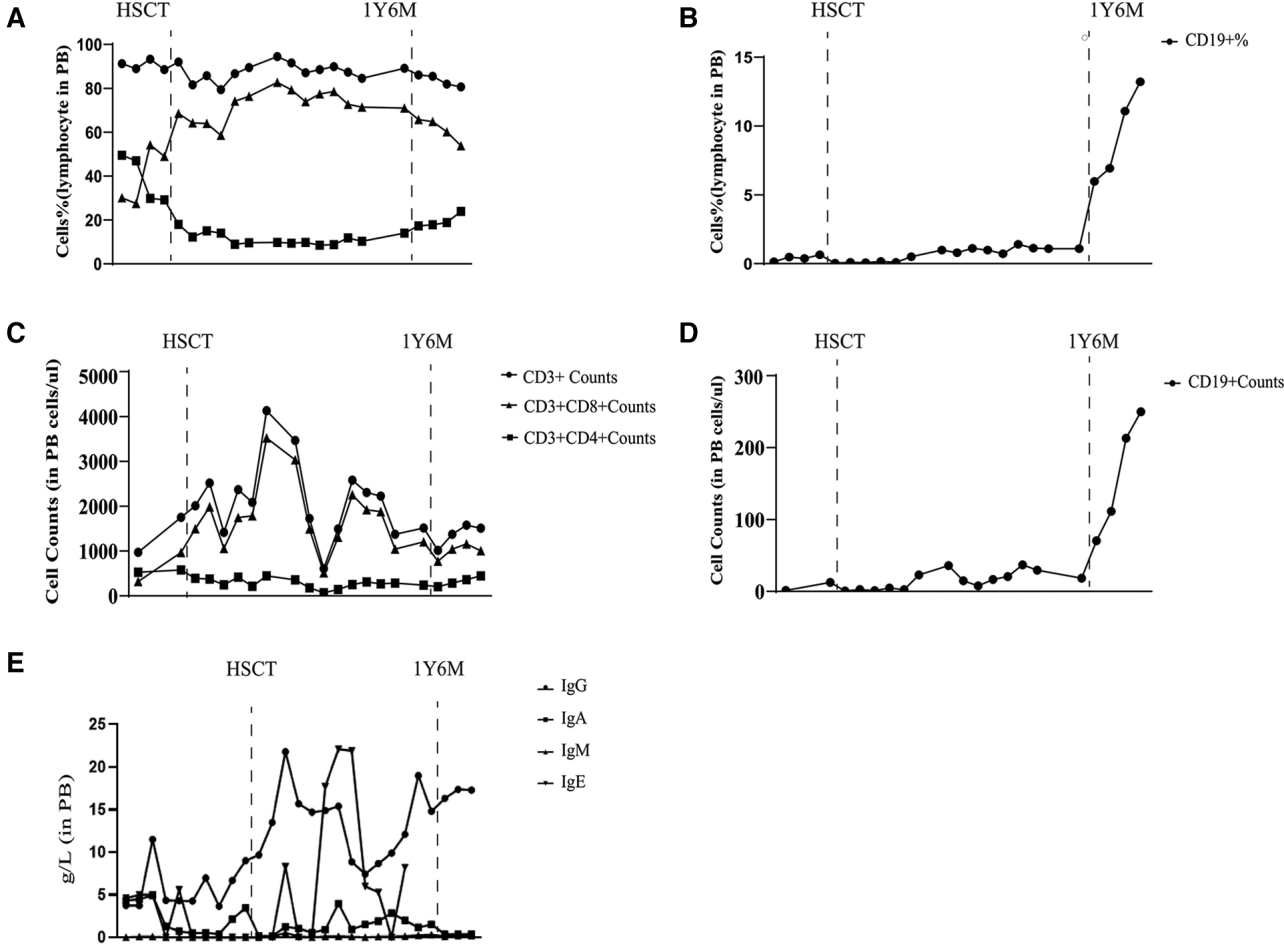
Laboratory values from diagnosis to one and a half years after transplantation. Proportion of CD3+, CD8+, and CD4+ T lymphocytes (**A**) Proportion of CD19+ B lymphocytes (**B**) Number of CD3+, CD8+, and CD4+ T lymphocytes (**C**) Number of CD19+ B lymphocytes (**D**) Immunoglobulin level (**E**).

## 4 Discussion

Lipopolysaccharide (LPS)-responsive vesicle trafficking and beach- and anchor-containing (LRBA) gene deficiency can lead to common variable immunodeficiency (CVID), which presents symptoms similar to autoimmune lymphoproliferative syndrome (ALPS) and manifestations reminiscent of X-linked immune dysregulation-nolvendocrinopathy, enteropathy (IPEX)-like syndrome. The specific treatment guidelines for CVID remain unknown, and symptomatic treatment is the main treatment. Current findings indicate that patients with LRBA deficiency exhibit dramatic and sustained improvement in response to abatacept, a CTLA4 (cytotoxic T lymphocyte antigen-4)–immunoglobulin fusion drug (1, 2). However, in this patient, abatacept did not respond well.

The indications for allogeneic haematopoietic stem cell transplantation (allo-HSCT) or gene therapy, such as severe combined immunodeficiency (SCID), are well-established. However, not all immunodeficient patients have clear indications for early allo-HSCT. Compared with novel immune system-dysregulative PIDs, these PIDs exhibit heterogeneous disease manifestations and lack genotype–phenotype correlations.

A study has demonstrated that patients undergoing hematopoietic stem cell transplantation have comparable survival probabilities to those treated with abatacept. While almost all LRBA-deficient patients respond positively to abatacept therapy, the extent of response varies among individuals with different clinical presentations. The highest rates of complete remission are observed in those with lymphoproliferative disorders, followed by those with chronic diarrhea. Conversely, responses to abatacept treatment for immunodeficiency-related symptoms display greater heterogeneit (7). In our patient's case, although there was a notable improvement in lymphoproliferative manifestations, recurrent infections persisted as the most concerning aspect throughout the course of the illness and were unaffected by Abatacept therapy. Consequently, after identifying a suitable donor through the Chinese Bone Marrow Bank, the patient and their family decided to proceed with hematopoietic stem cell transplantation for further treatment.

Several reports have documented the diagnosis of approximately 12 children with LRBA deficiency who underwent haematopoietic stem cell transplantation. Typically, these patients exhibit remission of clinical symptoms post-transplantation or present with LRBA-associated manifestations, such as autoimmune thrombocytopenia, necessitating therapeutic intervention (8–13). In a recent multicentre follow-up of 76 patients with LRBA deficiency, 24 patients underwent HSCT. The overall survival rate after HSCT was 70.8%. Transplant-related mortality accounted for all nonspecific early deaths following the procedure. It is possible that HSCT is chosen as a last resort after many organ complications due to the early years of immature transplantation techniques and insufficient knowledge of LRBA defects, thus reducing the success rate of HSCT (4). In addition, a survey study from Turkey on 15 children with LRBA deficiency revealed that among the seven who underwent HSCT, they were alive and showed positive outcomes (median duration: 2 years; range: 1–3 years) (14).

In this study, the patient had persistent chronic inflammation and cumulative organ damage, leading to the selection of HSCT as a treatment option due to its potential effectiveness in suppressing the disease process. At present, after one and a half years post-transplantation, significant improvements have been observed in the patient's condition with no apparent signs of infection, restored liver function, disappearance of nervous system manifestations, and normalization of immune cell populations. These findings indicate the efficacy of transplantation treatment; however, it should be noted that early-stage clinical manifestations following transplantation can be complex. This complexity may not solely arise from the transplant procedure itself but also pose considerable challenges due to primary lesion accumulation.

Due to autoinflammation leading to multiple organ complications, the patient experienced recurrent pulmonary infection before transplantation, resulting in evident lung fibrosis that exacerbated the transplant procedure's difficulty. The patient continued to experience recurrent pulmonary infections during the early stages of transplantation, likely due to abnormal lung function caused by pulmonary fibrosis and incomplete immune recovery post-transplantation. During the preparation for transplantation, the patient developed septic shock due to sepsis and diarrhoea, which improved after gamma globulin and anti-infection therapy with advanced antibiotics, but it became more difficult to use the conditioning regimen before transplantation.

It is worth noting that neurological changes are a major challenge of transplantation for this patient. As previously reported, LRBA deficiency patients may experience neurological manifestations primarily due to autoinflammatory disease, characterized by granulomatous brain lesions, nerve demyelination and atrophy, as well as occasional intracranial haemorrhages (15). Prior to transplantation, although the patient did not exhibit any neurological symptoms, the patient's brain MRI showed abnormal signals in the bilateral temporal lobe, right parietal lobe, and corpus callosum. Subsequently after transplantation, the patient developed subdural effusion caused by adenovirus infection and linear enhancement of the bilateral frontoparietal and occipital meninges, and then brain MRI revealed multiple abnormal signals in the bilateral white matter. Posttransplant leukoencephalopathy was diagnosed, likely due to the process of transplantation itself, but it cannot be ruled out that this was a step related to LRBA deficiency. Fortunately thereafter no evident changes were observed on MRI scans and no significant neurological findings emerged.

Immune reconstitution after transplantation in immunodeficient patients is also a major concern. We found that the patient's LRBA protein and CTLA4 had recovered to normal levels one and a half years after transplantation. However, regarding immune reconstitution, there was a relatively delayed recovery of B-cell function in the child following transplantation, possibly attributed to the utilization of a reduced-intensity conditioning regimen. However, regarding immune reconstitution, there was a relatively delayed recovery of B-cell function in the child following transplantation, possibly attributed to the utilization of a reduced-intensity conditioning regimen. Futhermore, glucocorticoids and immunosuppressants for the treatment of GVHD can inhibit the production of B lymphocytes as well. Previous studies have shown that B-cell reconstitution is faster in children with WAS syndrome than in children with CGD after transplantation (16). Therefore, whether LRBA deficiency itself has an effect on the reconstitution of B cells remains unknown and necessitates extensive research efforts. However, comparing pre- and post-transplantation B cell counts revealed that allogeneic haematopoietic stem cell transplantation played an important role in restoring lymphocyte levels among children with LRBA deficiency.

The use of HSCT for LRBA deficiency has demonstrated remarkable efficacy. However, the presence of complex disease activity, prolonged duration prior to HSCT, and irreversible organ damage are all associated with unfavorable outcomes. Seidel's team proposed (4) the IDDA scoring method, which was introduced by Seidel's team for evaluation and can further strengthen clinical management and be used to help doctors decide whether and when to perform HSCT in patients with LRBA deficiency.

Therefore, we believe that transplantation is an effective treatment for patients with multiorgan complications, but it is important to avoid a greater disease burden, longer duration before HSCT, and multiorgan involvement that increase the incidence of post-transplantation complications, which may reduce the chance of successful transplantation.

In conclusion, these data show that allogeneic haematopoietic stem cell transplantation could be considered a treatment option for individuals with LRBA deficiency. Nevertheless, more cases are needed to establish guidelines for the management of LRBA deficiency before and after transplantation.

## Data Availability

The raw data supporting the conclusions of this article will be made available by the authors, without undue reservation.
